# Greening Wine Exports? Changes in the Carbon Footprint of Spanish Wine Exports

**DOI:** 10.3390/ijerph18179035

**Published:** 2021-08-27

**Authors:** Inmaculada Carrasco, Juan Sebastián Castillo-Valero, Carmen Córcoles, Marcos Carchano

**Affiliations:** 1 Institute of Regional Development, University of Castilla-La Mancha, 02071 Albacete, Spain; inmaculada.carrasco@uclm.es (I.C.); Sebastian.Castillo@uclm.es (J.S.C.-V.); 2 Faculty of Economics, University of Castilla-La Mancha, 02071 Albacete, Spain; carmen.corcoles@uclm.es

**Keywords:** international trade, wine, carbon footprint, multi-regional input–output model

## Abstract

Spain is one of the leading wine-producing and -exporting countries and has traditionally been dominant in trade and world production in the sector. In an increasingly changing context, in which worldwide wine exports are growing exponentially, it is essential to study their impact on climate change as the transport of goods generates a significant volume of greenhouse gas emissions. The aim of this work, then, was to analyse the variation in the carbon footprint generated by Spanish wine exports between 2011 and 2016. To this end, a multi-regional input–output (MRIO) model was used, showing that the emissions associated with wine operations have increased less than exports, which might suggest that sustainable growth has been included as a goal in the wine supply chain. The methodology used has the advantage of allowing the calculation of direct and indirect emissions. At the same time, the results can provide relevant information to practitioners and policymakers due to the expected evolution of European environmental regulations and trades, in terms of carbon footprint.

## 1. Introduction

Wine production is a highly important activity in the global agriculture sector [1,2,3,4] from both an economic viewpoint and a social, cultural and environmental perspective. Its significance lies not only in the economic value it generates or the number of workers it employs, but it also stands out for the key role it plays in conserving the environment (capturing CO_2_ and stabilising ecosystems), population settlement and local development, especially in regions where there are scant economic alternatives [5,6,7]. Hence, this sector contributes to integrated territorial development, which is characterised by economic growth, inclusive governance, social cohesion and environmental sustainability [8].

In Spain, the wine industry is of great importance, being one of the primary “Old World” producers [9], with a vineyard area of 95,282 hectares and a production of 33.5 million hL in 2019 [10], making it the first country in vineyard-planted area and third in volume of production. Spain thus enjoys a privileged position in the international wine economy, but the Spanish wine sector is facing a difficult challenge due to the imbalance between supply and demand generated by the fall in internal demand over recent decades. For this reason, a growing share of the annual production is sold in the international market [11], where the main competitors are, on the one hand, France and Italy (also traditional “Old World” wine-producing countries), and, on the other, China, Australia, Chile, Argentina, the United States and New Zealand, all “New World” wine producers that have entered the international market with innovative production and marketing models. In this highly competitive and uncertain scenario, Spain is positioned as one of the leading wine exporters, ranked second in volume and third in value, with Spanish wine products being part of an immensely globalised supply chain [9].

Wine has traditionally been considered a “green” product [12] that respects the environment [13,14], leading to the wine sector paying scant attention to environmental problems [14,15]. However, non-organic wine production, which requires the use of fertilisers, water, pesticides and energy, among others, has a negative impact on the environment [15,16,17,18,19,20,21,22,23,24,25]. From a broader perspective, the agri-food sector (which includes wine) has an evident effect on the environment, as agriculture exploits about 38% of Earth’s terrestrial surface, consumes 70% of the water dedicated to human use and is responsible for about 29% of total greenhouse gas emissions [26,27]. In short, the wine industry, which benefits from natural resources and ecosystems (public assets), incurs costs and externalities, which are transferred to society [19].

In this sense, it is guessed that the wine sector contributes to environmental decline which is one of the main causes of global warming and climate change [28,29]. Currently, climate change is one of the biggest challenges facing human society. For that reason, its effects are being closely studied by researchers [30,31,32,33]. The emission and concentration of greenhouse gases, where CO_2_ represents 76% of the total, is a major contributor to climate change [34,35,36,37]. Specifically, the CO_2_ emissions contribute to the increase in temperature through the generation of greenhouse gases [38,39], which drain the ozone layer, causing a higher penetration of solar radiation [40]. As negative effects derivative of the greenhouse gases’ concentration, we must add air pollution, rise in sea level, environmental damage and ocean pollution, among others [41,42,43,44,45,46]. Consequently, many studies have emerged with the purpose of analysing the causes of CO_2_ increase, where the link between rise of emissions and economic growth [32,42,47,48,49], international trade, degree of commercial openness [49,50,51,52], degree of urbanization [53,54,55,56] and financial development are analysed [57,58,59,60]. As a result of this, the necessity of reducing the emissions of pollutant gases has become one of the main worldwide worries on the subject of sustainable development, as it is proved by Paris’ Agreement of 2015 or the climate strategy of EU 2016, which have as objectives to enhance the reduction in greenhouse gases in the economy by 20% in 2020 and 30% in 2030, compared with 1990. As concrete measures for this aim, we can highlight regulations of emissions decrease [61,62], carbon tax [63,64] or mechanisms of right emissions trade [32,65].

All the above, together with the increasing pressure from stakeholders to improve environmental performance, has led many wine companies to integrate social and environmental objectives into their strategy [66,67,68,69], forcing them to develop and implement new environmental practices and technologies [70], advancing towards environmentally sustainable cultivation and production practices [71,72,73,74]. Furthermore, consumer interest in the environmental profile of wines is growing [75,76,77,78,79,80,81], showing a certain predisposition to pay higher prices for these kinds of products [13,75,82,83], leading to pressure on companies to disclose their environmental performance [84]. In this way, the environmental profile is incorporated as an important element in the purchase decision [85,86,87]. Additionally, the increase in consumer preferences for sustainable wine products plays an important role in the winery differentiation strategy [88], so more and more are integrating these practices into their activity [89]. At the same time, this favors both the implementation of sustainable practices [77] and the innovation process [90]. Particularly, the adoption of practices oriented to a reduction in the carbon footprint can lead to sustained competitive advantages [91,92].

As a consequence, the concept of environmental sustainability (which combines environmental, economic and social aspects [90]) has gained importance in the sector [72,93,94,95], emerging as a key goal for all those that form part of the wine supply chain [14,67,69,75,92,96]. Greater awareness of questions of sustainability in the wine sector has led to the proliferation of protocols and instruments intended to promote sustainability in the industry [97,98].

In the context of a growing trade in wine products, international commerce is emerging as a primary contributor to the expansion of greenhouse gas emissions [99]. In the same line, Wiedmann and Lenzen [100] highlight that as much as 64% of global environmental impacts may be linked to international trade, with these effects growing in significance [101]. The international movement of goods and services implies greater transport of freight, with such transport becoming a major source of generation of greenhouse effect gases, accounting for 14% of global emissions [102]. Drawing on Colman and Päster [103] and focusing on the wine sector, transport is the part of the wine supply chain that produces the greatest impact on the environment; specifically, of the possible means of transport, the aeroplane is the most polluting, followed by the lorry, car, train and ship [104]. In this sense, such is the growth of international trade and, by extension, of transport, that, according to the Intergovernmental Panel on Climate Change (IPCC) [36], greenhouse gas emissions are expected to have increased by 50% in 2035, with this percentage being even higher by 2050. More alarming data are reported by Xu and Dietzenbacher [105], who indicate that while carbon dioxide emissions generated by production increased by 32% in the period 2005–2007, overall global emissions grew by 80% in the same period. Thus, the extensive impact of international trade on greenhouse gas emissions and consequently on the environment is more than evident. International trade puts great pressure on the agricultural industry, with croplands for export production increasing 2% year on year between 1986 and 2009 [106].

The main aim of the present study is to analyse the changes in the carbon footprint of wine exports in the period 2011–2016, the latest years for which all the data required for the model used are available. To this end, a multi-regional input–output model was used, enabling us to calculate the carbon footprint associated with Spanish wine exports. The carbon footprint is an environmental indicator that measures the greenhouse gas emissions directly or indirectly generated by production and consumption [107,108,109]. For our case study, carbon footprint has special relevance, as it is considered a good indicator to help improve environmental performance in this sector [110,111].

Numerous authors have estimated the wine sector’s carbon footprint [103,112,113,114,115,116,117,118,119]. Most of these authors, however, focus on life cycle analysis, centring on the production and vinification of a bottle of wine or on bulk wine production. The calculation of the carbon footprint associated with the international wine trade has, to the best of our knowledge, been the subject of considerably less attention in the literature.

## 2. International Trade in Spanish Wine

**Figure 1:**
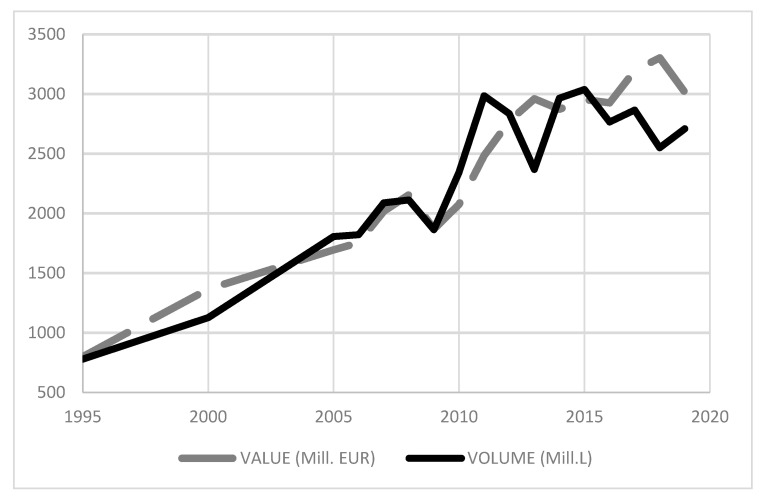
Variation in wine exports, 1995–2019.

The globalisation process has triggered significant changes in the world’s wine markets, affecting both “Old World” and “New World” producing countries. In this process, Spain, as a producer from the first group, has witnessed an increase in the volume and value of sales to the foreign market since the middle of the 20th century, a trend that has continued to today. In recent years, wine marketed abroad has doubled that consumed within Spain (see Figure 1).

Since 1995, the first year for which data are available, Spanish wine exports have multiplied by five in both value and volume, growing from a little more than 500 million litres to 2690 million litres in terms of value and 2124 million litters in terms of volume. Nonetheless, the evolution of volume and value of wine exports has had ups and downs over the years. At the end of the 20th century, growth, which had stagnated since 1980 due to the prevailing economic crisis, picked up again, continuing until 2011, with a downward trend that lasted until 2013, mainly as a result of a lower volume of production. 

This situation turned around in 2014 and 2015 as a consequence of the good harvest in 2013. The growth path that restarted in those years has continued until today, although the year-on-year increase has been more moderate. This situation can be extended to the analysis of export value, with the exception of recent years, in which a reduction has been noted due to a fall in average prices, leading to a lower volume of turnover. Specifically, in absolute terms, in 2019, approximately 130 million more litres of wine were exported compared to the previous year, but sales fell by 234 million euros due to a 20-cent fall in the average price. Recent developments in exports show a high degree of variability, especially in volume, with an increase of almost 30% in 2011 and a drop of 20% in 2013. In addition, these oscillations are less dramatic in the most recent years under study, due to the reduction in the imbalance between wine production and sales (supply problem).

In short, as discussed, the evolution of wine exports has varied greatly, as a result not only of production in the national market but also of production in world markets. In this sense, if internal production is large and external production small, it is to be expected that the countries producing less resort to importing wine to compensate for a temporary fall in internal production.

## 3. Materials and Methods

### 3.1. Multi-Regional Input-Output (MRIO) Model

The methodology proposed for this study is the multi-regional input–output model, which allows us to capture the economic relationships between regions and sectors under study. This model, the standard framework for which was developed by Miller and Blair [120], enables the assessment of environmental and social pressures in the global economy [100,121]. However, the use of this methodology introduces several limitations. On the one hand, the input–output model is expressed in monetary terms, so that they are not considered directly the physic units. Additionally, the wine sector is approximated through the sector of “manufacture of food products, beverages and tobacco products”, which is the one that has a more similar productive structure. Despite certain limitations, many authors consider it an ideal method for the analysis of environmental, social and economic impacts [107,122].

The multi-regional model includes all the different countries’ intersectoral relationships; that is, every country is included considering its own technology and trade (intermediate and final) for each good. The model draws on the basic equation of the classic input–output model, which can be expressed as follows:

(1) x=Ax + y

where x is the total production, A is the matrix of technical coefficients and y is the final demand (wine exports, in our case), but the MRIO model, as stated, includes all the intersectoral relationships of the different countries. Thus, the matrix structure for the basic equation of the multi-regional model for m countries is as shown below (2):

(2) $$\left(\begin{array}{c} \begin{array}{c} x^{1}\\ x^{2}\\ \ldots \\ x^{m} \end{array} \end{array}\right)=\left(\begin{array}{cccc} A^{11} & A^{12} & \ldots & A^{1m}\\ A^{21} & A^{22} & \ldots & A^{2m}\\ \ldots & \ldots & . & \ldots \\ A^{m1} & A^{m2} & \ldots & A^{\mathrm{mm}} \end{array}\right)*\left(\begin{array}{c} x^{1}\\ x^{2}\\ \ldots \\ x^{m} \end{array}\right)+\left(\begin{array}{c} y^{1}\\ y^{2}\\ \ldots \\ y^{m} \end{array}\right)$$

Specifically, given that our aim is to measure the environmental impact of wine exports, we need to incorporate the carbon emissions into our model, and the export vector is considered in the final demand (y). These emissions are incorporated using the emission coefficient (e_i_), which is defined as the emissions generated by sector “i” for the production of one unit of product, where E represents the emissions generated in the production activities.

(3) $$e=\frac{E}{x}$$

Multiplying each member of the basic input–output model equation by the emission coefficient and the final demand, we obtain:

(4) $$F=\;\hat{e}\;{\left(I-A\right)}^{\left(-1\right)}\;\hat{y}=P\hat{y}$$

where (^) expresses the diagonalization of the vector, and P = $\hat{e}\;{\left(I-A\right)}^{\left(-1\right)}$ is the resulting emission multiplier, which measures the direct and indirect impacts of each sector and country per unit of product in each sector. The generic matrix structure for the emission multiplier for two countries and two sectors can be represented as follows:

(5) $$\left(\begin{array}{ccccc} \varepsilon_{11}^{11} & \varepsilon_{12}^{11} & \vdots \; & \varepsilon_{11}^{12} & \varepsilon_{12}^{12}\\ \varepsilon_{21}^{11} & \varepsilon_{22}^{11} & \vdots \; & \varepsilon_{21}^{12} & \varepsilon_{22}^{12}\\ \ldots & \ldots & \vdots \; & \ldots & \ldots \\ \varepsilon_{11}^{21} & \varepsilon_{12}^{21} & \vdots \; & \varepsilon_{11}^{22} & \varepsilon_{12}^{22}\\ \varepsilon_{21}^{21} & \varepsilon_{22}^{21} & \vdots \; & \varepsilon_{21}^{22} & \varepsilon_{22}^{22} \end{array}\right)\;=\;\left(\begin{array}{cc} p^{11} & p^{12}\\ p^{21} & p^{22} \end{array}\right)$$

where ε ^rs^_ij_ shows the emissions of sector “i” from country “r” to satisfy a unit of final demand of sector “j” from country “s”.

Thus, observing matrix P by rows, we have the emissions resulting from the production process of a good, while the columns show the emissions incorporated into the production process through the inputs used in manufacturing the product. 

In our case, we multiply the diagonalized wine export vector (y), following the example above (two countries, two sectors) and by columns we obtain the emissions generated by these exports.

(6) $$\left(\begin{array}{cc} p^{11} & p^{12}\\ p^{21} & p^{22} \end{array}\right)*\left(\begin{array}{c} y^{11}\\ 0 \end{array}\begin{array}{c} 0\\ y^{22} \end{array}\right)=\left(\begin{array}{ccccc} p^{11} & y^{11} & \vdots \; & p^{12} & y^{22}\\ \ldots & \ldots & \vdots \; & \ldots & \ldots \\ p^{21} & y^{11} & \vdots \; & p^{22} & y^{22} \end{array}\right)$$

### 3.2. Database

To calculate the environmental impact of Spanish wine exports for the period 2011–2016, we used three datasets: data provided by the most recent version of the World Input-Output Database (WIOD) [123], from which we obtained the input–output tables for 44 regions and 56 sectors (available up to 2014. For the 2016 calculations, we used the WIOD data for 2014 (the most recent available) under the assumption that there were no structural changes in the sector); data taken from the same source on emissions expressed in kilotons of CO_2_; and finally, the data provided by the Spanish Wine Market Observatory [124] on the value of wine exports in millions of euros at sale price. The results of the current work are presented using the same units as in the data provided (Mill. USD and KtCO_2_). The technical coefficients are those of the agri-food sector. The calculations were performed using the latest version of MATLAB.

## 4. Results and Discussion

**Figure 2:**
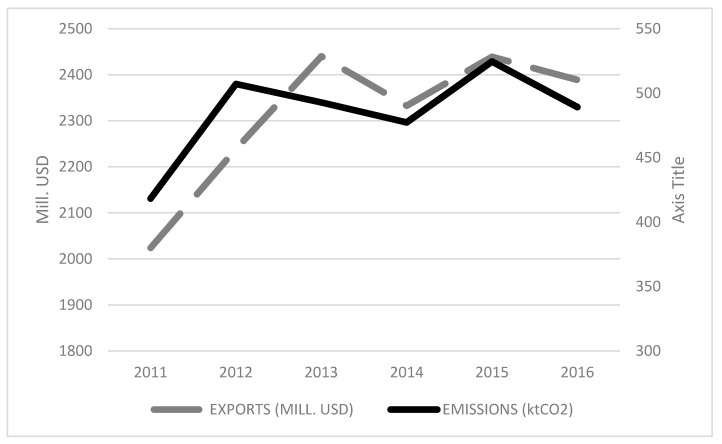
Recent variations in exports (Mill. USD) and emissions (ktCO_2_), 2011–2016.

For the in-depth study of the consumption-based emissions, we focused on the recent variations in the carbon footprint of wine exports in the period 2011–2016 (see Figure 2). A close relationship can be observed between the evolution of exports by value (Mill. USD) and emissions (KtCO_2_), as both variables moved in the same direction and underwent the same changes, although with differing intensity across the years. The emissions increased more sharply than exports in the first period under study (2011–2012), and, from 2012, the emissions showed negative growth rates, while exports grew steadily. This situation continued until 2014, when the volume and values of exports both increased due to the good harvest of 2013–2014, which generated a rise in the CO_2_ emissions. Finally, in 2015, a slight decrease in emissions can be observed. In short, although in the later years a certain decrease in the carbon footprint of Spanish wine exports can be observed, this rose from 418.2 KtCO_2_ in 2011, to 489.2 KtCO_2_ in 2016, with the peak being reached in 2015 (524.5 KtCO_2_).

**Figure 3:**
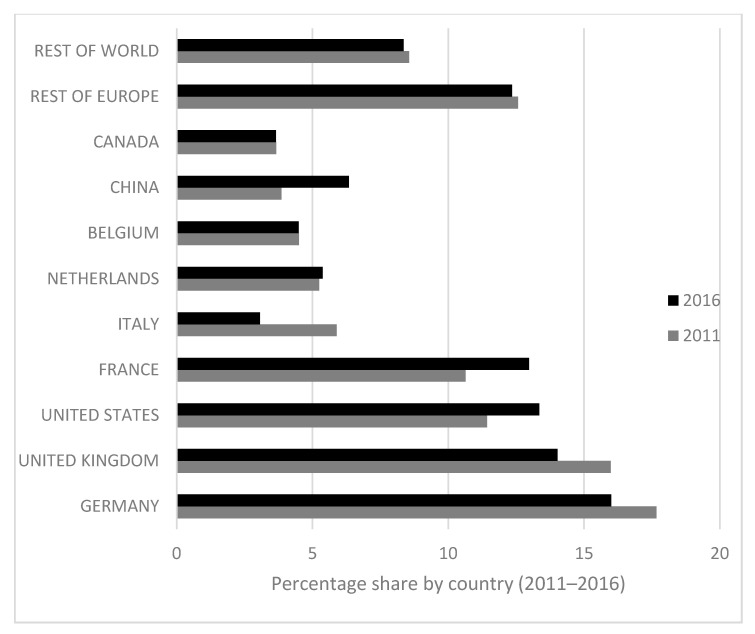
Percentage share by country of the carbon footprint generated by Spanish wine exports (2011–2016).

The percentage share of each country in the emissions generated by Spanish wine exports (Figure 3) has also changed noticeably as a result of changes in Spain’s trade partners. In this sense, European “Old World” countries, such as Germany, Italy and the United Kingdom, contributed less to the carbon footprint of Spanish wine exports, while the percentage share of France, China and the United States grew. In 2011, the emissions generated by exporting to China and the United States accounted for 4% and 11%, respectively, of the overall wine export carbon footprint. However, these amounts increased by around 2% between 2011 and 2014 to stand at shares of 6.8% and 13.4%. Despite the changes that took place in this period, Germany, the United Kingdom and the United States continue to be the countries where the environmental impact of Spanish wine exports is greatest, together accounting for approximately 40% of total CO_2_ emissions. The largest share of the remaining 60% corresponds to France (63.4 ktCO_2_), China (31.1 ktCO_2_), the Netherlands (26.3 ktCO_2_) and Belgium (21.8 ktCO_2_). Of the other countries, notable contributors are Canada and Italy, with 4% and 3%, respectively. 

**Figure 4:**
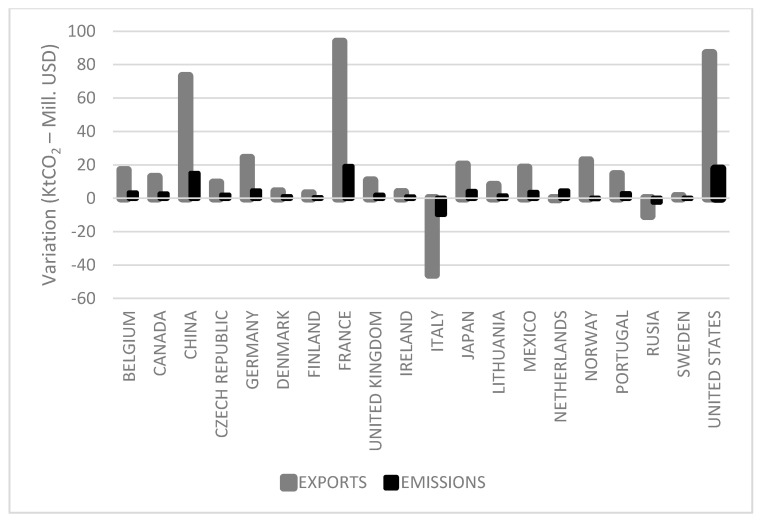
Variation of emissions (ktC0_2_) and exports (Mill. USD) (2011–2016).

Finally, Figure 4, which shows the changes recorded between 2011 and 2016 for emissions and economic value of Spanish wine exports (Mill USD) by country, highlights the differences in the variations between both variables. Although exports and emission moved in the same direction, they varied in intensity. Taking the specific case of Germany, for example, it can be seen that the emissions derived from these sales grew at a lower rate than the value of their imports between 2011 and 2016. This pattern is repeated for all the other countries except the Netherlands, suggesting that more sustainable methods of distribution were used, since, despite the growing amount of trade, the increase in emissions was proportionally lower in most of the countries. Consequently, it can be said that sustainable growth has emerged as an objective of the wine supply chain [14,75], driving companies to develop and implement new environmentally friendly behaviours [70].

In short, the analysis conducted in this study corroborates the findings of Santiago-Brown et al., (2015), in the sense that the concept of sustainability has progressively gained a foothold in the wine sector over recent years, and, more specifically, in the distribution process, which accounts for 22% of the sector’s total emissions [125]. However, despite the advances made in the fight against climate change in the wine sector, the growth in international trade over the last decade may tarnish these results, as the escalation of these types of operations has a negative impact on the environment, especially in this sector [16,17], which, in turn, incurs cost and externalities that are transferred to society [19]. Consequently, we cannot ignore the relative significance of the wine sector in the annual carbon footprint generated by global anthropogenic activity (0.3%) [125].

## 5. Conclusions

Wine production is one of the principal economic activities in a number of Spanish and European regions; in the specific case of Spain, it accounts for approximately 1.5% of gross value added. In recent years, questions pertaining to climate change and sustainable development have had a major impact on the wine sector, giving rise to cultivation and production practices that respect the environment. 

The internal demand for wine in Spain has fallen over the last twenty years, generating imbalances between supply and demand. While the supply of wine has remained practically stable, thanks to the contribution of the regions of Castilla-La Mancha, Catalonia and Extremadura, which make up 60% of national production, the demand has dropped by more than 30% since the beginning of the 21st century, accounting now for just a quarter of current production. This oversupply has forced Spain and other European countries, such as France and Italy, to resort to international markets to provide an outlet for their wines. In the specific case of Spain, in recent years, the volume of wine sold abroad has doubled that destined to internal consumption. Thus, the international market has emerged as the solution for the Spanish wine sector to maintain the level of activity recorded until now. 

In light of the importance of foreign trade for the Spanish wine sector and its environmental consequences, the aim of the present work was to study the development of the carbon footprint of Spanish wine exports in the period 2011–2016. For this purpose, a multi-region input–output model was used, by which we were able to quantify the overall environmental impact of these wine exports and how they are shared among the countries under study. In this regard, Germany, the United Kingdom, France and the United States are the countries that most contribute to the emissions generated by the export of Spanish wine products, while Belgium, China, the Netherlands, Canada, Japan and Italy account for a lower share of such emissions.

The main contribution of this work lies in demonstrating the direct relationship between exports and emissions generated by the Spanish wine sector. In addition, it shows that the emissions associated with trade in wine have increased less than the exports, which might suggest that sustainable growth has been adopted as an objective in the wine supply chain.

The results show that the wine sector is aligned with the goal of achieving a circular economy and carbon neutrality for 2050, promoted by Spain and EU governments. Knowing the carbon footprint of exports will allow the sector to make a more informed defense of price linked to the potential cost of permissions in the EU’s Emissions Trade System (the EU’s carbon market). Certificates would be per ton of CO_2_ emissions from imported products. This new system, which the EU plans to impose, will have to be compatible with that established by the Trade Mundial Organisation (TMO), but it will probably affect the multilateral relationships in the international wine trade. 

Finally, we must indicate that, in spite of limitations, the methodology used has the advantage of allowing us to calculate the direct emissions as well as the indirect ones, and the carbon footprint would be able to be defined for imported products such as fertilisers, one of the basic aspects on which the European Commission focuses to establish a potential adjustment in border for trade of products. Additionally, as it is a multiregional model, it provides us with sectorial information disaggregated by country, which indicates the total requirements of each sector and in each country to produce exported wine as well as the carbon footprint generated for each of them. In this sense, relevant lines of future research are opened, such as the viability to establish voluntary carbon markets for the exchange of environmental credits.

## Data Availability

Not applicable.
